# Early patellofemoral articular cartilage degeneration in a rat model of patellar instability is associated with activation of the NF-κB signaling pathway

**DOI:** 10.1186/s12891-021-03965-8

**Published:** 2021-01-18

**Authors:** Wei Lin, Huijun Kang, Yike Dai, Yingzhen Niu, Guangmin Yang, Jinghui Niu, Ming Li, Fei Wang

**Affiliations:** Department of Orthopedic Surgery, Third Hospital of Hebei Medical University, No. 139 Ziqiang Road, 050051 Shijiazhuang, Hebei China

**Keywords:** Patellar instability, Patellofemoral osteoarthritis, NF-κB signal pathway

## Abstract

**Background:**

Patellar instability (PI) often increases the possibility of lateral patellar dislocation and early osteoarthritis. The molecular mechanism of early articular cartilage degeneration during patellofemoral osteoarthritis (PFOA) still requires further investigation. However, it is known that the NF-κB signaling pathway plays an important role in articular cartilage degeneration. The aim of this study was to investigate the relationship between the NF-κB signaling pathway and patellofemoral joint cartilage degeneration.

**Methods:**

We established a rat model of PI-induced PFOA. Female 4-week-old Sprague-Dawley rats (*n* = 120) were randomly divided into two groups: the PI (*n* = 60) and control group (*n* = 60). The distal femurs of the PI and control group were isolated and compared 4, 8, and 12 weeks after surgery. The morphological structure of the trochlear cartilage and subchondral bone were evaluated by micro-computed tomography and histology. The expression of NF-κB, matrix metalloproteinase (MMP)-13, collagen X, and TNF-ɑ were evaluated by immunohistochemistry and quantitative polymerase chain reaction.

**Results:**

In the PI group, subchondral bone loss and cartilage degeneration were found 4 weeks after surgery. Compared with the control group, the protein and mRNA expression of NF-κB and TNF-ɑ were significantly increased 4, 8, and 12 weeks after surgery in the PI group. In addition, the markers of cartilage degeneration MMP-13 and collagen X were more highly expressed in the PI group compared with the control group at different time points after surgery.

**Conclusions:**

This study has demonstrated that early patellofemoral joint cartilage degeneration can be caused by PI in growing rats, accompanied by significant subchondral bone loss and cartilage degeneration. In addition, the degeneration of articular cartilage may be associated with the activation of the NF-κB signaling pathway and can deteriorate with time as a result of PI.

## Background

Osteoarthritis (OA) is a chronic joint disease that affects more than 100 million people worldwide [1, 2]. Usually, OA most strongly affects the knee joint, a three-compartment structure that includes the patellofemoral joint (PFJ) and the medial and lateral tibiofemoral joints. Previous studies on knee OA have focused on the tibiofemoral joints, and little research has been done on the PFJ [2]. However, isolated patellofemoral osteoarthritis (PFOA) is not only common but has a high prevalence in men (18.5–19.0 %) and women (17.1–34.0 %) aged 55–60 years [3, 4]. Furthermore, PFOA is the main cause of anterior knee pain and disability [5].

The PFJ is a unique and complex structure [6], which is stabilized by a complex multivariate relationship of osseous joint geometry and force vectors generated by the capsuloligamentous stabilizers and quadriceps femoris [7]. Patellar instability (PI) is frequently caused by pathological changes involving PFJ stabilizers, which increase the likelihood of lateral patellar dislocation and early OA [8]. Additionally, PI is related to trochlear dysplasia [8]. Schottle et al. demonstrated that trochlear dysplasia of the femur is a predisposing factor for recurrent PI [9]. Huri also demonstrated that PI is related to trochlear dysplasia [10]. In the general population, the incidence of primary PI is 5.8 per 100,000, with a higher incidence in more active and younger individuals [11]. Some risk factors, such as lateralization of tibial tuberosity, trochlear dysplasia, and the medial patellofemoral ligament, are believed to promote PI [12]. Biomechanical investigations have suggested that the medial patellofemoral ligament plays a crucial role in restraining lateral patellar translation and provides 50–60 % of PI; the retinacular fibers and patellomeniscal ligament were also demonstrated to be significant medial stabilizers. It is speculated that these ligament structures can provide proprioceptive signals for surrounding muscle tissues in addition to their biomechanical properties [13]. Although the PFJ plays an important role in knee OA, studies on the pathogenesis of PFOA are scarce [14]. The spontaneous PFOA model is characterized by slow disease progression, long study duration, and frequent changes in outcome [15]. A rat model of joint inflammation consists of intra-articular injection of monosodium iodoacetate that chemically induces chondrocyte death, but may not be a representative model of OA [16, 17]. The surgically induced OA model is mainly used to study tibiofemoral osteoarthritis, with only few studies on PFOA [18–20]. Additionally, the influence of PI on PFOA remains unclear.

It is well known that collagen X [21, 22] and MMP-13 [23], markers of cartilage degeneration, lead to chondrocyte imbalance and matrix degradation. Compared with normal cartilage, the cartilage of OA patients exhibits up-regulated TNF-ɑ levels [24]. Recently, the NF-κB signaling pathway was found to play an important role in regulating inflammatory mediators associated with OA [25]. This pathway serves as a bridge between cartilage degeneration and development, and few studies have investigated the role of PFOA in early cartilage degeneration.

Therefore, we investigated the relationship between cartilage degeneration during PFOA and the NF-κB signaling pathway by employing immunohistochemistry to assess the histological characteristics of cartilage and quantitative real-time polymerase chain reaction (qRT-PCR) to assess the different expressions of NF-κB, MMP-13, collagen X, and TNF-ɑ in cartilage at different time points in a growing rat model of PI.

## Methods

### Study design

**Fig. 1:**
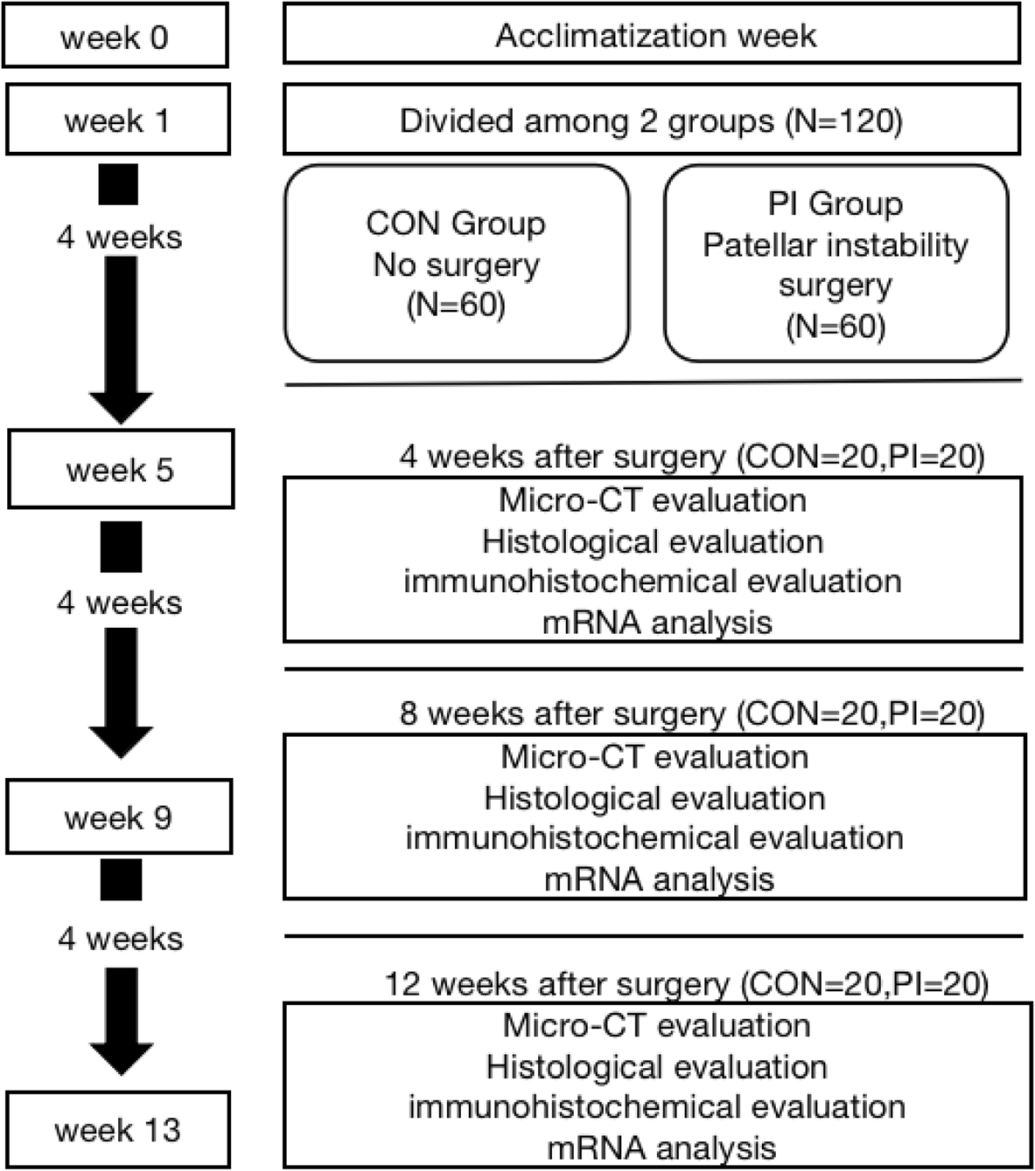
Schematic of the experimental protocol

This study was approved by the Animal Ethics Committee of the Third Hospital of Hebei Medical University (approval number: Z2019-005-1). Female 4-week-old Sprague-Dawley rats (50–80 g) were provided by the Laboratory Animal Center of Hebei Medical University. Rats (*n* = 120) were randomly divided into two groups: the control (*n* = 60) and PI group (*n* = 60). In the control group, the rats’ left knees did not undergo any surgery. In the PI group, the rats’ left knees underwent surgery to induce PI. Afterwards, left distal femur tissues were collected (*n* = 20 knees/time point in each group) (Fig. 1). All the rats were sacrificed by an overdose of anesthesia at 4, 8, and 12 weeks after surgery. According to animal euthanasia guidelines [26, 27], rats were intraperitoneally injected with an overdose of pentobarbital sodium (200 mg/kg). Rats were placed in a controlled environment (60 ± 5 % humidity, 12/12 h light/dark cycle, 22 ± 3 °C) and were free to obtain food and water. The animals were accustomed to the laboratory environment with an adaptation period of 1 week before experimentation.

### Surgical technique

**Fig. 2:**
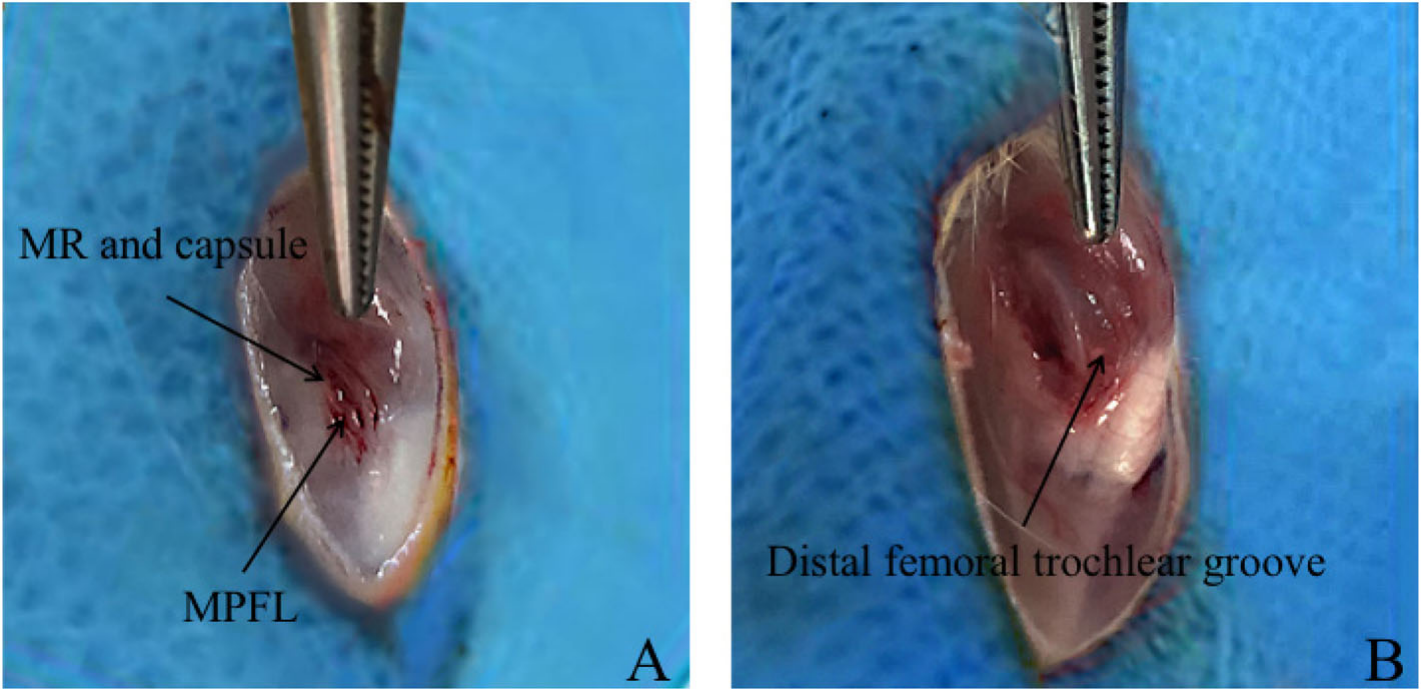
Detailed procedure of the operations: **a**, Cut off the medial soft tissue restraints (MPFL: medial patellofemoral ligament, MR: medial retinaculum); **b**, Postoperative PI (Patellar instability). Distal femoral groove can be noticed after the release of medial soft tissue restrains

Before surgery, pentobarbital sodium (30 mg/kg, intraperitoneal injection) was used to induce anesthesia in all rats; the left knee was placed in an extended state. After the surgical area was shaved, the surgery was prepared aseptically. A midline skin incision was performed; after the skin and subcutaneous tissue were separated, the capsule was exposed through the medial approach of the knee. Similar methods of surgery-induced PI have been reported in our previous studies [28, 29]. In this study, the medial retinaculum of the knee joint was cut off, and PI could be observed during surgery. The incision was then closed without reconstruction of the medial patellofemoral ligament, retinaculum, or capsules. After surgery, dressing was applied (Fig. 2). For post-operative care, animals were provided with 24 h quiet recovery time in a warm and dry environment. They were also provided with food to promote gastrointestinal peristalsis and to prevent stagnation after fully awakening. To control post-operative pain, rats were administered acetaminophen (30 mg/kg, daily) for 5 days.

### Micro‐computed tomography (micro-CT) assessment

**Fig. 3:**
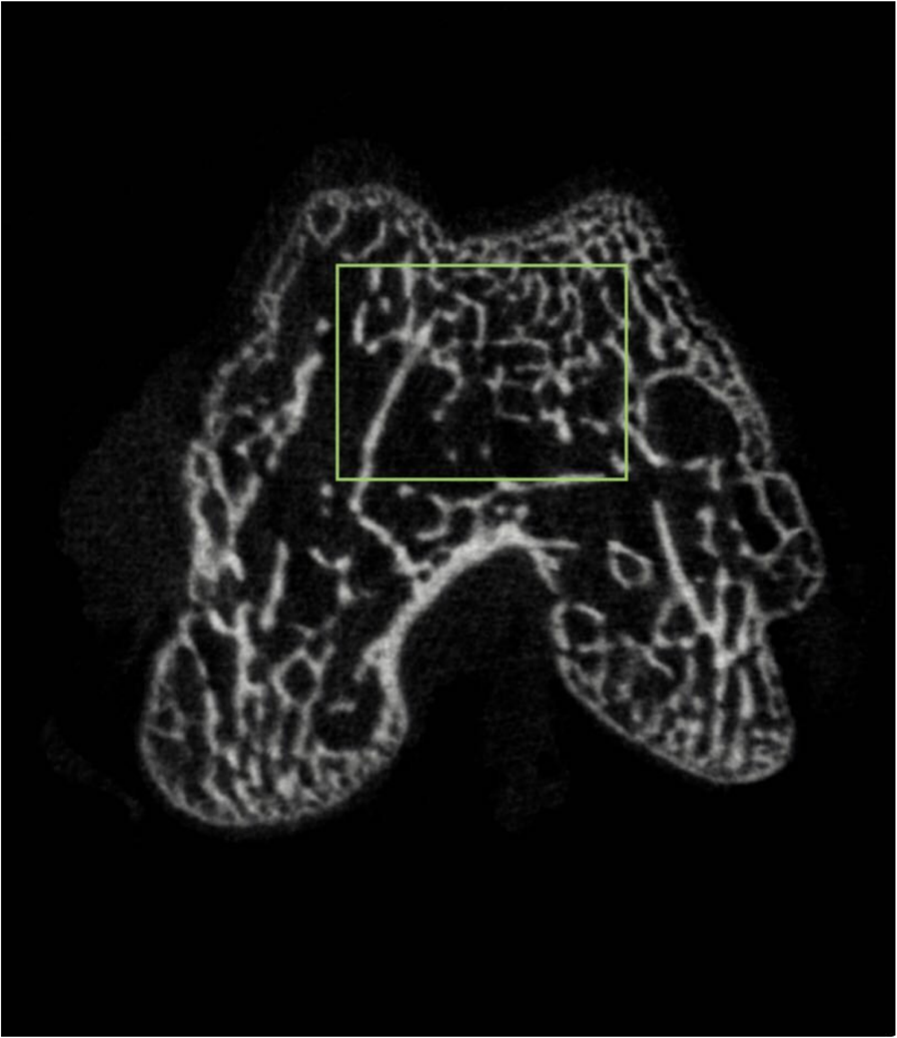
Axial view of the distal femur. ROI was shown within the green rectangular area. (ROI: region of interest)

In order to assess PI-induced changes in the subchondral bone microstructure, the bone tissues of distal femurs were carefully harvested and scanned via a micro-CT (SkyScan model 1076, Skyscan, Belgium) at 4, 8, and 12 weeks after surgery (n = 5 knees/time point in each group). All scans were performed with a voxel size of 10 µm at 50 kV and 800 µA. The region of interest (ROI) was located transversely below the trochlea with a green rectangle (4 × 3 mm) (Fig. 3). Based on previous studies, the following representative parameters were selected to assess changes in subchondral bone [2]: bone volume to total volume fraction (BV/TV fraction), bone mineral density (BMD), trabecular (TB) thickness, TB separation (SP), structure model index (SMI), TB pattern factor (PF), TB number, and degree of anisotropy (DA).

### Histology

The bone tissues of distal femurs were carefully harvested at each time point (n = 5 knees/time point in each group), fixed with 4 % polyformaldehyde, decalcified in 10 % EDTA until completely demineralized, and embedded in paraffin. Sections (5 µm) were cut along the femoral axis to obtain the transverse images of the trochlea sulcus. Safranin O was used to evaluate cartilage glycosaminoglycans. Cartilage degradation was evaluated by subjecting protein to fast green counter staining and scored using a semi-quantitative histopathological grading system based on the Osteoarthritis Research Society International (OARSI) score [30, 31]. After observation with a microscope, a camera was used to acquire images of the representative sections. Chondrocyte numbers were quantified in the superficial and middle zone and expressed as numbers/mm^2^. Values were calculated from at least five nonconsecutive sections per region.

### Immunohistochemistry

The slices were deparaffinized in xylene, rehydrated, and washed three times for 5 min with phosphate buffered saline at room temperature. Endogenous peroxidase activity was blocked by 3 % hydrogen peroxide (H_2_O_2_) for 10 min. Antigen retrieval was performed by microwave treatment in 10 mm of sodium citrate (pH 6.0) for 10 min. This was followed by overnight incubation at 4 ℃ with anti-collagen X (BoAoSen, BeiJing, China), anti-MMP-13 (Servicebio, WuHan, China), anti-TNF-ɑ (ABclonal, WuHan, China), and anti-NFKP65 (Servicebio, Wu Han, China) at a dilution of 1:50. The primary antibody was omitted from the negative control reaction. Afterwards, images were acquired with a 20 × 100 objective lens from five randomly selected areas from each slice in each group (n = 5 knees/time point in each group). The tissue filled the entire field of view, and the background light of each photo was the same. Image-Pro Plus 6.0 software (Media Cybernetics, Rockville, MD, USA) was used for image analysis and choose the same brown color as the uniform standard for judging all positive photos. Data regarding integrated optic density of positive cell and the pixel area of tissue were acquired for all images. The areal density was defined as the integrated optic density divided by the pixel area of tissue and was positively correlated with positive cell expression.

### qRT-PCR

At 4, 8, and 12 weeks after surgery, the cartilage was peeled off from the trochlear with a blade and was analyzed by qRT-PCR for mRNA expression (n = 5 knees/time point in each group). Trizol reagent (Servicebio, Wuhan, China) was used to extract RNA from the cartilage. mRNA was translated into cDNA by the RevertAid™ first strand cDNA synthesis kit (Thermo, #K1622) according to manufacturer’s instructions. The target genes were MMP-13, collagen X, TNF-ɑ, and NF-κB p65; all genes were analyzed by applying the sequence detection system, and the expression of related mRNA was standardized relative to the GAPDH gene and calculated with the 2^−ΔΔCt^ (cycle threshold) method. The primers used are shown in Table 1. We performed each experiment three times and obtained the average values.

**Table 1:** Primers used for amplification of target genes and GAPDH

	Forward primer sequence	Reverse primer sequence
Collagen X	CCCTTTCTGCTGCTAGTGTCCT	GGAATGCCTTGTTCTCCTCTTAC
MMP-13	TGCATACGAGCATCCATCCC	CGTGTCCTCAAAGTGAACCGC
TNF-ɑ	CCACCACGCTCTTCTGTCTACTG	TGGGCTACGGGCTTGTCACT
NF-κB p65	CTTCACTCGGAGACTGGAACCT	AAATCCTTCCCAAACTCCACC
GAPDH	CTGGAGAAACCTGCCAAGTATG	GGTGGAAGAATGGGAGTTGCT

### Statistical analysis

All data values were expressed as mean ± standard deviation. Data were analyzed with the statistical software GraphPad Prism (GraphPad software V7, La Jolla, CA, USA).The Shapiro-Wilk test was used to evaluate normality, the Levene’s test was used to evaluate the homogeneity of variance, and two-way analysis of variance was used to evaluate the parametric data between the two groups at each time point. *p* < 0.05 was considered statistically significant. According to the results of the preliminary experiments, each group and each time point required at least six rats, with a confidence of 90 % and an efficacy of 80 % (1 − β) [32].

## Results

### Micro-CT measurements of subchondral bone

**Fig. 4:**
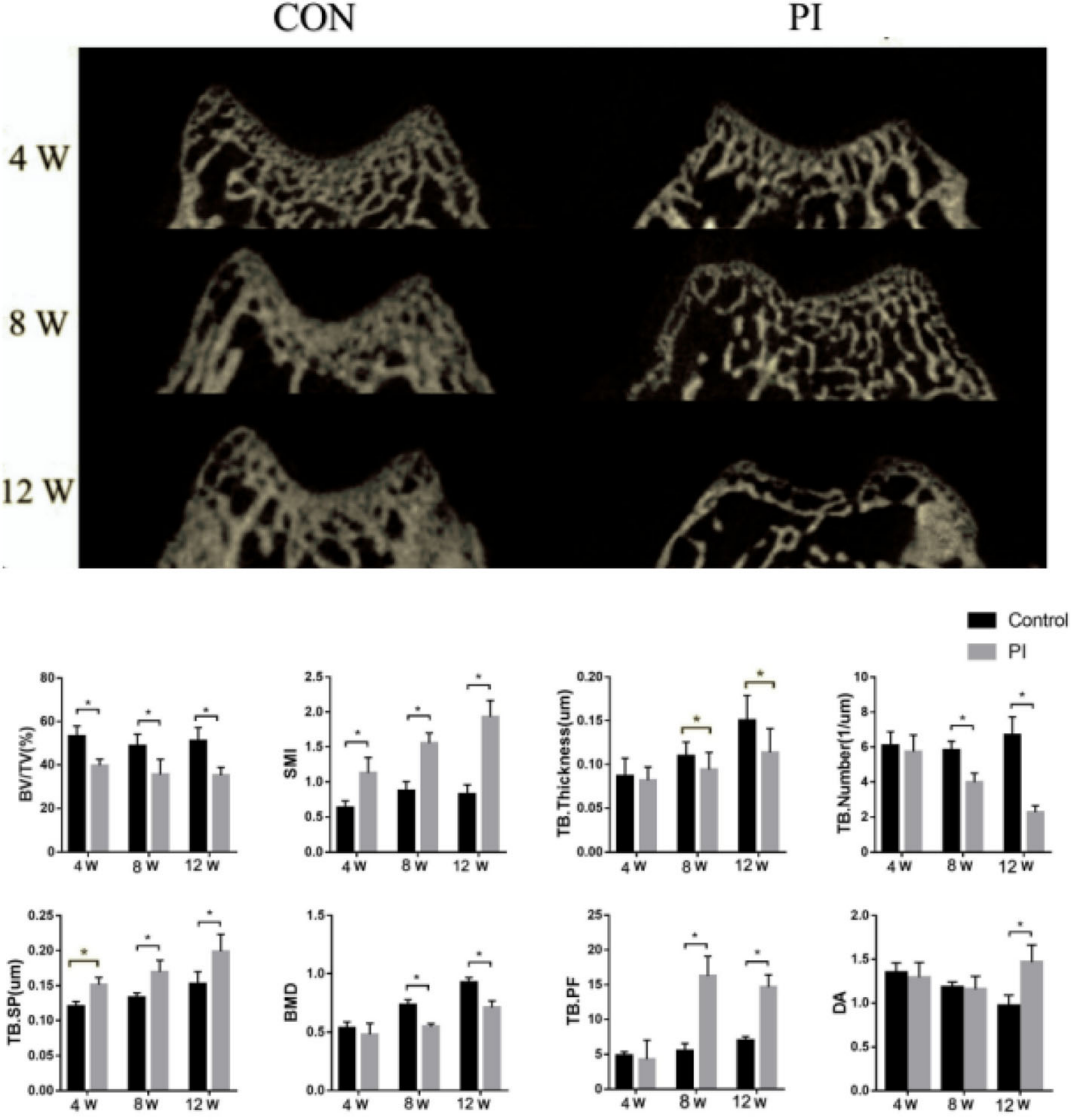
Micro-CT showed the configuration of femoral trochlear and the subchondral bone in the CON and PI groups at all times points. In the PI group, the center of the trochlear groove become flat, significantly bone loss was observed at 8,12 weeks after surgery, and the loss of bone mass gradually aggravated. Architectural parameters of subchondral bone samples of different groups. *P < 0.05 (BV/TV: bone volume to total volume; BMD: bone mineral density; TB: trabecular; SP: separation; SMI: structure model index; PF: pattern factor; DA: degree of anisotropy; CON Group, control group; PI Group, patellar instability group)

In the PI group, significant bone loss was observed at 4 weeks compared with the time-matched controls; the loss of bone mass gradually increased, and the subchondral bone plate became obviously thinner (Fig. 4). Micro-CT revealed that the BV/TV fraction decreased (*p* < 0.05), while the SMI and TB SP increased 4 weeks after surgery (*p* < 0.05) (Fig. 4). Compared with the time-matched controls, TB thickness, TB number, and BMD decreased (*p* < 0.05), while the TB PF increased 8 and 12 weeks after surgery (*p* < 0.05) (Fig. 4).

### PI leads to articular cartilage degeneration

**Fig. 5:**
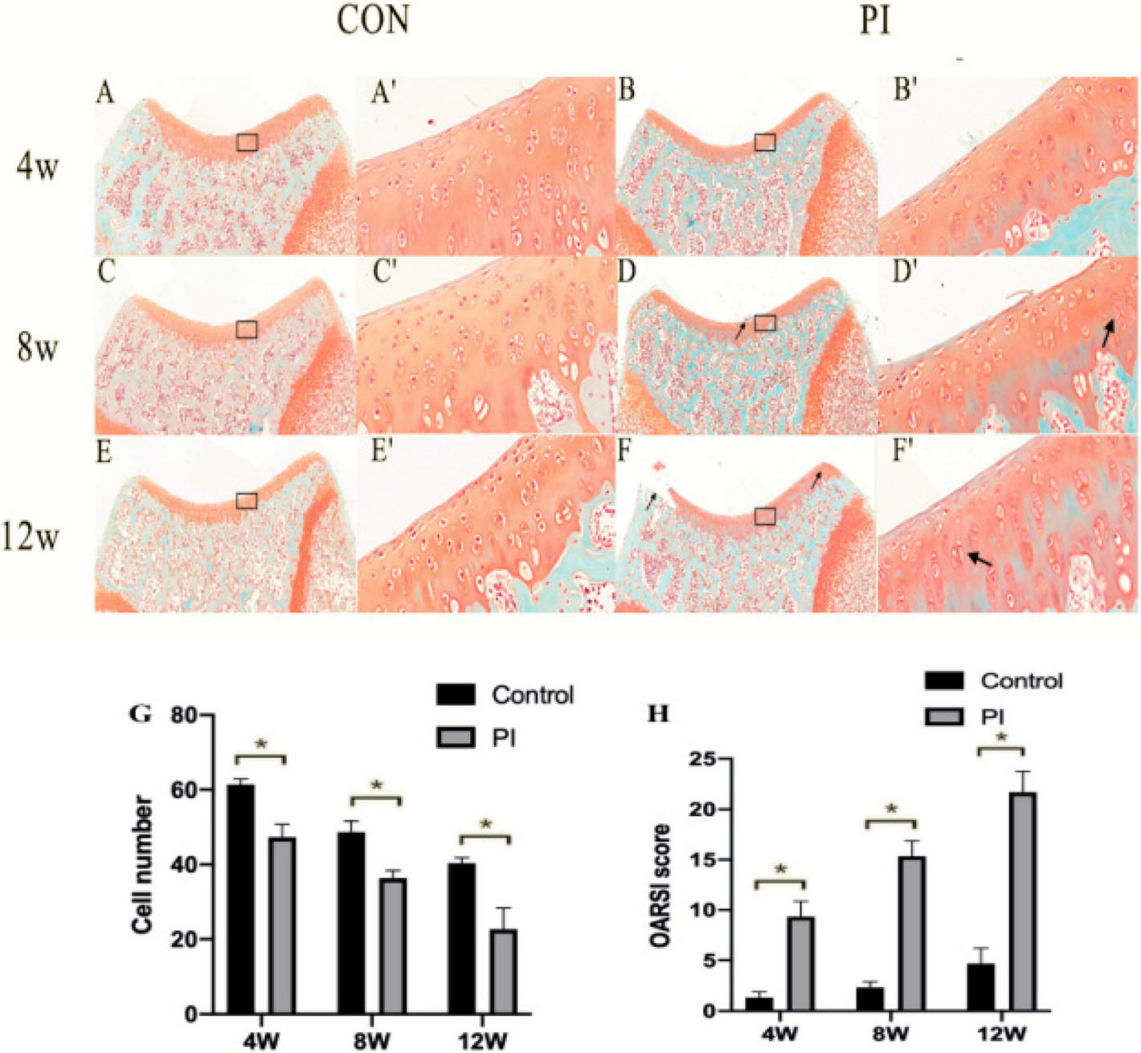
SafraninO and fast green staining in cartilage of rats. CON and PI group respectively: **a**, **b** 4 weeks; **c**, **d** 8 weeks; and (**e**, **f**) 12 weeks. (**A′**–**F′**) images at a higher magnification of boxed areas in (**a**–**f**). In the PI group, safranin O and fast green histochemical staining showed that the surface of the cartilage was irregular and destroyed at 8, 12 weeks after surgery(the thin black arrow in **d**, **f** ). In **D′**, **F′**, we observed cell aggregation(the thick black arrow), and the number of chondrocytes in the superficial and middle zone was significantly reduced, which was consistent with OA. **g** estimating cell numbers of the superficial and middle zone; **h** OARSI score for articular cartilage: increased OARSI score and decreased cell number were observed in the PI Group at 4,8, and 12 weeks after surgery. *p < 0.05 (CON Group, control group; PI Group, patellar instability group)

Four weeks after surgery, we determined the relative positive staining area and the structural changes of the cartilage surface layer. We observed cell aggregation, loss of cartilage structure, and the Safranin O staining reduced significantly consistent with OA, 8 and 12 weeks after surgery (Fig. 5). In the PI group, safranin O and fast green histochemical staining showed that the cartilage surface was eroded and irregular and that the number of chondrocytes in the superficial and middle zone was significantly reduced (*p* < 0.05) 12 weeks after surgery (Fig. 5). However, in the control group, the chondrocytes were flattened and the cartilage surface was smooth at different time points. Compared with the control group, higher OARSI scores were observed 4, 8, and 12 weeks after surgery in the PI group (*p* < 0.05) (Fig. 5).

### PI increased the expression of MMP-13, collagen X, TNF-ɑ, and NF-κB P65

**Fig. 6:**
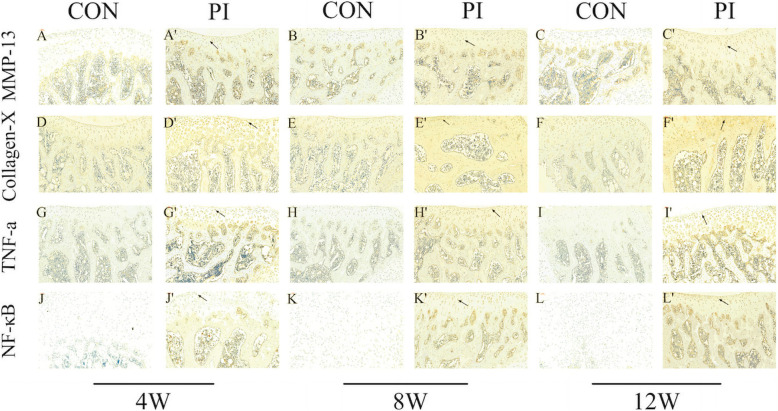
Results of immunohistochemical analysis for the two groups: MMP-13 (CON group: **a**–**c**; PI Group: **A’** –**C’** ); Collagen X (control group: **d**-**f**; PI Group: **D’** –**F’** ); TNF-ɑ (control group: **g**–**i**; PI Group: **G’** –**I’** ); NF-κB (control group: **j**-**l**; PI Group: **J’** –L’ ). Higher expression of MMP-13 (**A’** –**C’** ), Collagen X (**D’** –**F’** ), TNF-ɑ (**G’** –**I’**), and NF-κB( **J’** –**L’**) were observed in the PI Group at 4, 8, and 12 weeks after surgery. The black arrows in (**A’**-**L’**) indicate positive expression and cell cluster formation. A brown color indicates positive staining. Scale bar: 50 µm (CON Group, control group; PI Group, patellar instability group)

**Fig. 7:**
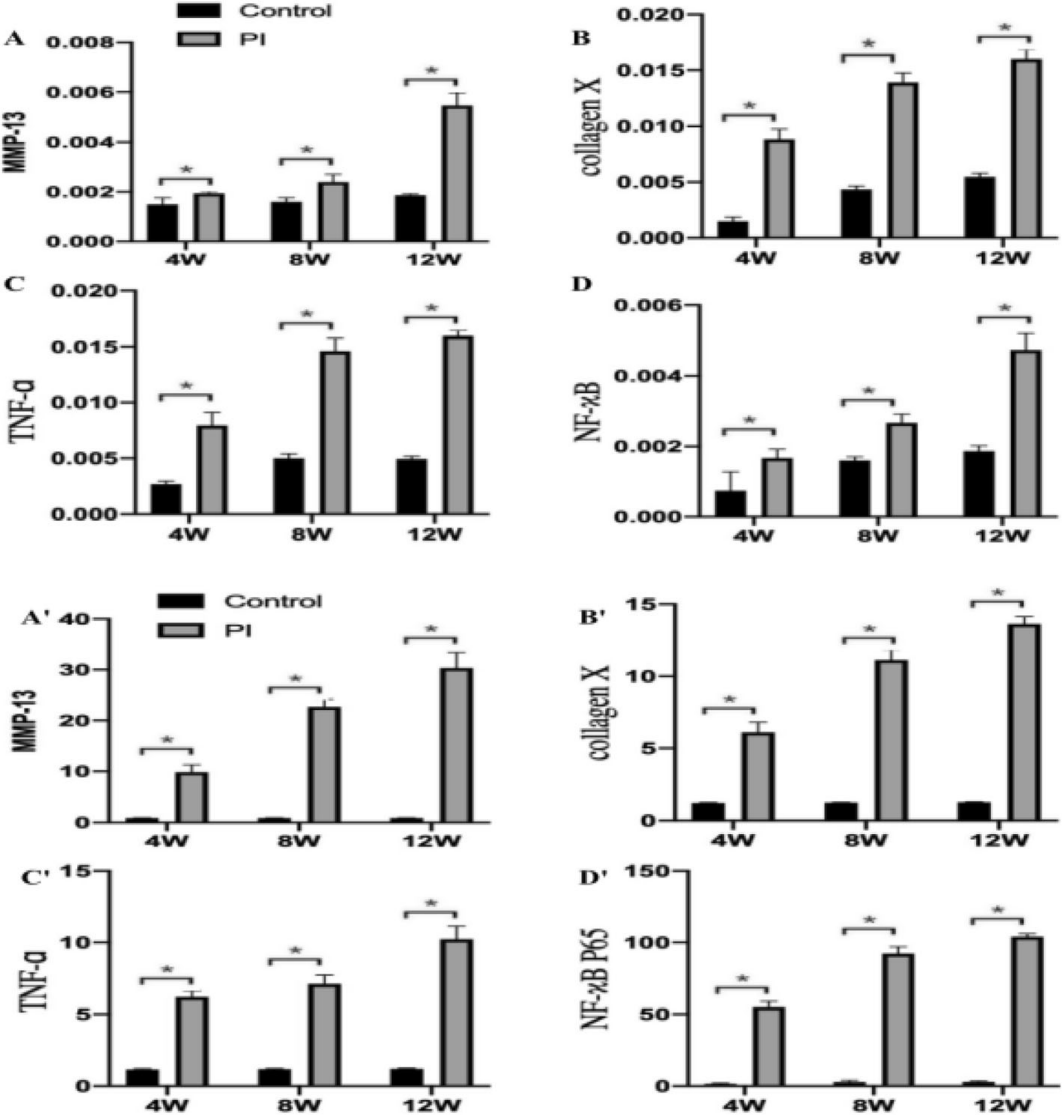
The areal density of immunoistochemical analyses (**a**-**d**) and target gene expression of qRT-PCR evaluation ( **A′**-**D′**) in the two groups: showed higher expression of MMP-13, Collagen X, TNF-ɑ, and NF-κB in the PI Group at 4, 8, and 12 weeks after surgery. *p < 0.05 (PI Group, patellar instability group)

Immunohistochemical analysis revealed that there were significant differences in MMP-13, collagen X, TNF-ɑ, and NF-κB p65 4, 8, and 12 weeks after surgery (*p* < 0.05) (Fig. 6). Furthermore, qRT-PCR analysis revealed expression patterns similar to those of the immunohistochemical analysis (*p* < 0.05) (Fig. 7).

## Discussion

In this study, we successfully established a rat PI-induced PFOA model and found that PI aggravated subchondral bone loss and cartilage degeneration, which worsened over time. Furthermore, we observed significantly elevated NF-κB protein and mRNA levels in the PFOA model, which indicates that PI-induced cartilage degeneration may be associated with activation of the NF-κB signaling pathway.

The present PFOA rat model exhibited obvious cartilage damage, which might be related to PI-induced patellofemoral hypertension and an abnormal load of PFOA-related structural damage [33]. One study demonstrated that mechanical stress is an important factor affecting cartilage and bone development [34]. PI-induced mechanical stress can destroy the integrity and dynamic balance of the PFJ. With the development of PI, the load in the PFJ redistributes, and the microstructure of the trabecular bone changes. The most obvious change was observed in the distal femur; the BV/TV fraction, TB thickness, TB number, and BMD decreased with time after surgery. This may be related to the abnormal pressure load that increase osteoclast activity, which may lead to a significant increase in osteoclast bone absorption in subchondral bone [35]. Furthermore, cartilage degradation was apparent from 4 weeks after surgery. In the PI group, we observed cell aggregation, loss of cartilage structure and the Safranin O staining reduced significantly at 8 and 12 weeks after surgery, this is consistent with human OA. The safranin O and fast green histochemical staining showed that the cartilage surface was eroded and irregular, the number of chondrocytes in the superficial and middle zone was significantly reduced at 12 weeks after surgery. However, in the control group, the chondrocytes were flattened and the cartilage surface was smooth at different time points. These results suggest that the changes in the microstructure and morphology of subchondral bone may be accompanied by gradual cartilage degradation and may further aggravate cartilage degradation during the process of PFOA [29].

The main feature of OA affecting the joint is the degeneration of articular cartilage. And, the altered phenotype of articular chondrocytes is the initial step in the progression of OA disease [36, 37]. In patients with osteoarthritis, MMP-13 is the main member of MMP family expressed in cartilage, but not in normal adults [38–40]. MMP-13 levels in patients with osteoarthritis indicate the degree of cartilage degeneration [38–40]. Activated MMPs catalyze the decomposition of cartilage matrix and induces chondrocyte apoptosis, leading to cartilage damage [41]. It has been found that MMP-13 can degrade collagen II in human OA [42]. Additionally, the reduced load decreases the proteoglycan content and causes cartilage thinning, which leads to cartilage degeneration [43]. Collagen X is a cartilage-specific collagen. Under normal circumstances, it is limited to the hypertrophic area of the growth plate, where it participates in endochondral ossification. Although Collagen X is not a component of normal articular cartilage, it is present in OA cartilage, especially in the deep regions where hypertrophic chondrocyte clusters are observed. Mutations in the Collagen X gene (COL10A1) lead to various forms of metaphyseal dysplasia [44]. These studies are consistent with our findings. In the present study,we found that the expression of MMP-13 and Collagen X in immunohistochemistry and RT-PCR level were significantly higher. We believe that PI can significantly reduce the pressure in the PFJ, resulting in cartilage degeneration. In conclusion, our findings support the view that articular chondrocytes of the PI model exhibit the same molecular phenotype as the early stage of OA.

The pathogenesis of OA is not completely clear; however, it is closely related to many inflammatory factors [45]. It has been confirmed that the levels of pro-inflammatory cytokines, such as TNF-ɑ and IL-1b, increase sharply during the early stages of OA progression. Animal and in vitro studies have confirmed that the inflammatory cytokine TNF-ɑ can induce bone resorption and enhance cartilage degradation in OA [46, 47]. In our study, we found higher immunohistochemical and mRNA expression of TNF-ɑ, which can stimulate the production of other cytokines, such as IL-6, prostaglandin, and MMP [46] as well as decrease type II collagen and proteoglycan levels [48]. This may cause PFJ cartilage degradation after PI.

It is generally believed that the NF-κB signaling pathway plays a significant role in regulating inflammatory mediators associated with OA pathogenesis [25]. NF-κB most likely combines with the inhibitory subunit IκBα and presents in the cytoplasm in an inactive form. Once induced by IL-1β, NF-κB activation requires sequential phosphorylation, degradation of IkBa, and NF-κB p65 translocates from the cytoplasm to the nucleus to induce the expression of inflammation-related genes, including TNF-ɑ, MMPs, COX-2, PGE2, NO, iNOS, IL-6, and ADAMTS [49, 50]. In addition to regulating TNF-ɑ and IL-1b expression, NF-κB is also activated by these cytokines [51], thus forming a vicious circle. In our study, immunohistochemistry and mRNA expression analyses revealed that NF-κB levels increased over time in the PFOA model of PI. Thus, persistent high expression of NF-κB in the PFOA model may explain the persistent existence of developmental factors in articular cartilage. However, in this study,we did not investigate the phosphorylation of NF-κB, so we only speculated that early patellofemoral articular cartilage degeneration in a rat model of PI is associated with activation of the NF-κB signaling pathway, further studies are needed to clarify how NF-κB contributes to PFOA pathology.

There are some limitations in our study. First, although this PFOA model cannot be translated to human OA, it can be used to study the therapeutic effects of drugs on OA. Second, because the shape of the patellar in rats is relatively small and difficult to obtain, we have not studied the changes of patellar cartilage degeneration. Third, we need to further study at the cellular level the molecular mechanism of the NF-κB signaling pathway in patellofemoral cartilage degeneration.

## Conclusions

A rat model of PI-induced PFOA was successfully established by studying changes in cartilage and subchondral bone. This animal model may be useful for future research on PFOA. In addition, the PI-induced degeneration of patellofemoral cartilage may be related to the activation of the NF-κB signaling pathway and may deteriorate with time.

## Data Availability

The detailed data and materials of this study were available from the corresponding author through emails on reasonable request.

## Abbreviations

PI: Patellar instability
PFOA: Patellofemoral osteoarthritis
OA: Osteoarthritis
PFJ: Patellofemoral joint
MMP-13: Matrix metalloproteinase-13
Micro-CT: Micro-computed tomographic
qRT-PCR: Quantitative real-time polymerase chain reaction
BV/TV: Bone volume to total volume
BMD: Bone mineral density
TB: Trabecular
SP: Deparation
SMI: Dtructure model index
PF: Pattern factor
DA: Degree of anisotropy
